# Mass and stiffness spectrometry of nanoparticles and whole intact bacteria by multimode nanomechanical resonators

**DOI:** 10.1038/ncomms13452

**Published:** 2016-11-11

**Authors:** O. Malvar, J. J. Ruz, P. M. Kosaka, C. M. Domínguez, E. Gil-Santos, M. Calleja, J. Tamayo

**Affiliations:** 1 Institute of Microelectronics of Madrid (IMM-CSIC), Isaac Newton 8 (PTM), Tres Cantos, 28760 Madrid, Spain; 2 Matériaux et Phénomènes Quantiques, Université Paris Diderot, CNRS, Sorbonne Paris Cité, UMR 7162, 10 rue Alice Domon et Léonie Duquet, Paris 75013, France

## Abstract

The identification of species is a fundamental problem in analytical chemistry and biology. Mass spectrometers identify species by their molecular mass with extremely high sensitivity (<10^−24^ g). However, its application is usually limited to light analytes (<10^−19^ g). Here we demonstrate that by using nanomechanical resonators, heavier analytes can be identified by their mass and stiffness. The method is demonstrated with spherical gold nanoparticles and whole intact *E. coli* bacteria delivered by electrospray ionization to microcantilever resonators placed in low vacuum at 0.1 torr. We develop a theoretical procedure for obtaining the mass, position and stiffness of the analytes arriving the resonator from the adsorption-induced eigenfrequency jumps. These results demonstrate the enormous potential of this technology for identification of large biological complexes near their native conformation, a goal that is beyond the capabilities of conventional mass spectrometers.

## Abstract (web summary)

Mass spectrometry can accurately identify species by molecular mass, but measuring large species can be difficult. Here the authors show that nanomechanical resonators can identify both the mass and stiffness of larger analytes, demonstrating it for gold nanoparticles and *E. Coli* bacteria.

## Graphical abstract

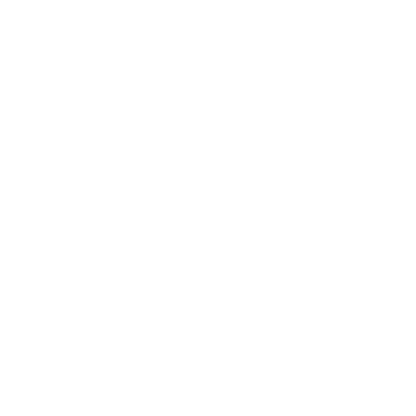

Mass spectrometry (MS) measures the mass-to-charge ratio of molecular species from 100 Da to 100 kDa with extremely high accuracy^1^,^2^. However, the performance largely degrades on the measurement of heavier species such as nanostructures and biological assemblies. In these cases, the charged analytes are successively fragmented by several dissociation methods causing a complex mass spectrum pattern that is not readily convertible into the original analyte. Nanomechanical resonators, such as singly and doubly clamped beams at the micro- and nanoscale, have recently emerged for measuring the mass of analytes with outstanding sensitivity^3^,^4^,^5^,^6^,^7^, ∼Da, and high dynamic range, enabling the measurement of nanostructures and large macromolecular complexes^8^. The physical principle is that the resonance frequency of the resonator is a sensitive function of its mass. The smaller the device, the more susceptible is the resonant frequency to minuscule added masses^9^,^10^. The unparalleled attributes of nanomechanical resonators have motivated a new paradigm of MS, referred to as nanomechanical spectrometry that enables the measurement of the mass of entire analytes with no need of charge state characterization^9^,^10^,^11^. In nanomechanical MS, the species are usually introduced by electrospray ionization (ESI) from the fluid phase into vacuum and are subsequently guided by ion optics to the resonator in high vacuum (<10^−5^ torr)^9^. Alternatively, matrix-assisted laser desorption ionization can be used for delivering the sample to the resonator^10^. As each analyte adsorbs on the mechanical resonator, abrupt resonance frequency downshifts are observed that are proportional to the ratio of the analyte mass to the device mass with a proportionality constant that depends on the adsorption position. The nanomechanical signature is insensitive to the charge of the adsorbate, simplifying the analysis of the data^11^. Deconvolution of the mass and adsorption position along the resonant beam requires the simultaneous measurement of at least two vibration modes^10^,^12^,^13^.

We recently developed a theory on the mechanical coupling between biological particles and resonant beams that predicts the analyte stiffness may significantly influence the resonance frequency shifts on particle adsorption^14^. This effect is particularly relevant when the particle thickness is comparable to the beam thickness and it depends on the elastic modulus and geometry of the particle, as well as on the interfacial adhesion between the particle and the resonator. However, this theoretical prediction has not been experimentally confirmed in nanomechanical spectrometry assays. In general, the stiffness effect is smaller and it can be easily hidden by the mass effect. Theoretical methods for extracting the mass of the particles landing on the resonator thus neglect the stiffness. This simplification also reduces the complexity of the inverse problem for deriving the particle mass and position from the resonance frequency jumps.

In this study, we perform nanomechanical spectrometry of 100 nm-sized gold nanoparticles (GNPs) and *Escherichia coli* DH5α cells using microcantilever resonators. We develop theoretical methods that enable the determination of the stiffness, mass and position of the analytes arriving the microcantilever from the resonance frequency jumps. Ignoring the effect of the stiffness leads to an underestimation of the mass of ∼10% for the used microcantilevers. More importantly, we estimate the Young’s modulus of the *E. coli* cells, which is consistent with the value obtained by atomic force microscopy (AFM).

## Results

### Nanomechanical spectrometer system

**Figure 1:**
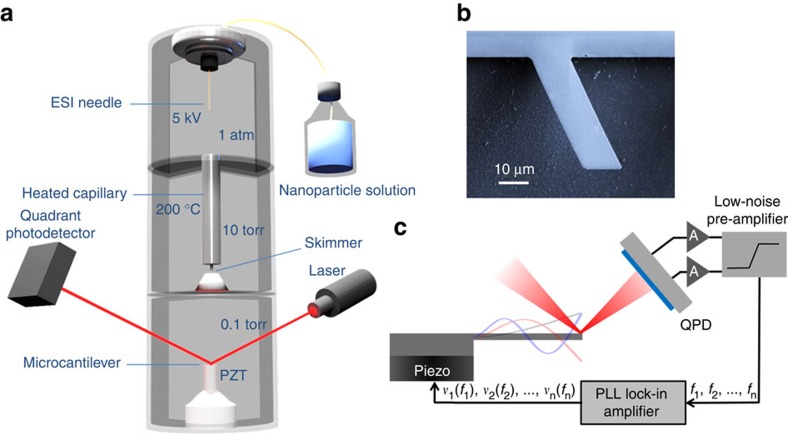
Nanomechanical spectrometer. (**a**) Schematic of the experimental set-up. It comprises three chambers with decreasing pressure from top to bottom: the ESI chamber at ambient pressure, the heated capillary chamber at 10 torr and the resonator chamber at 0.1 torr. The charged species are driven to the nanomechanical resonator through the pressure gradient. (**b**) SEM image of a 100 nm-thick silicon nitride microcantilever used in the experiments with GNPs. (**c**) Schematic of the optical beam deflection method used for measuring the cantilever vibration. The cantilever is excited by means of a piezoelectric actuator (piezo) beneath the cantilever chip. Digital PLLs are used to track in real time the resonance frequency of the first flexural vibration modes. A, current-to-voltage amplifier; *f*_*n*_=resonance frequency of *n*th mode; QPD, quadrant photodetector; *v*_*n*_, AC voltage signal at frequency *f*_*n*_ sent by the PLL to the piezoelectric actuator.

Figure 1a schematically depicts our prototype of nanomechanical spectrometer that comprises three stages with decreasing pressure. At the first stage, an ESI unit is used to generate mostly desolvated charged species at ambient pressure. The charged species are immediately attracted by the second stage, a heated metallic capillary at 200 °C and vacuum pressure ≈10 torr placed at 5 mm below the ESI needle. The elevated temperature aids to fully desolvate the microdroplets and to prevent analyte loss by sticking to the internal wall of the capillary. The charged particles at the exit of the heated capillary are attracted by a skimmer with a 100 μm-wide orifice that connects with the third stage, a low vacuum chamber (≈0.1 torr) in which the nanomechanical resonator is placed a few centimetres below the hole. The resonator (Fig. 1b) is driven by a piezoelectric actuator placed underneath and the vibration is detected by the optical beam deflection method^15^,^16^ (Fig. 1c). It is noteworthy to emphasize that the vacuum pressure of the resonator chamber is at least four orders of magnitude higher than in previous nanomechanical spectrometers. This moderately limits the attainable frequency resolution due to the fact that the quality factor is lower than in high vacuum^17^. However, it allows to place the nanomechanical resonator close to the ion source (at 18 cm in comparison with ∼2 m required in previous set-ups), which eliminates the need of ion guide optics. The proximity between the ion source and the nanomechanical detector is advantageous to achieve high capture efficiencies (one of the main limitations of both conventional^18^ and nanomechanical^9^,^10^,^11^ MS). In the case of GNP experiments, the ion beam at the detector chamber has a flow rate of ≈4 particles per second, a cross-section diameter of 600 μm at the resonator’s position and a low divergence of ≈0.2° (Supplementary Fig. 1 and Supplementary Discussion). The flow rate at the detector chamber represents ∼0.24% of the particles emitted by the ESI needle. If we multiply this number by the ratio of the resonator’s plan view area to the ion beam cross-sectional area, we obtain a capture efficiency of ≈10^−5^. Larger efficiencies approaching 100% can be readily envisaged by using two-dimensional arrays of cantilevers^15^ combined with nano-ESI ionization^19^.

### Theory of nanomechanical spectrometry

**Figure 2:**
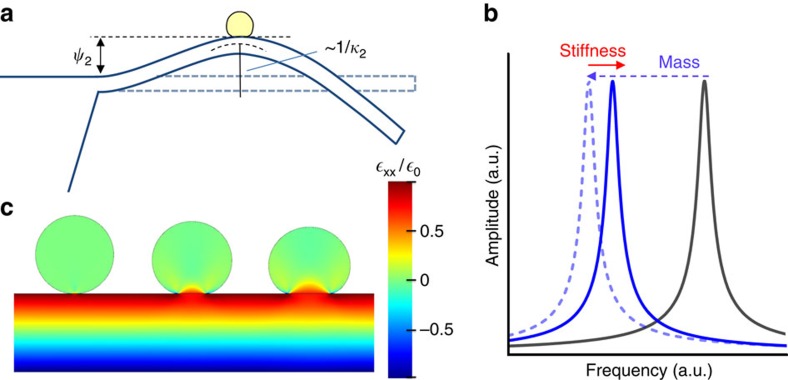
Effect of nanoparticle adsorption on the resonance frequency of a cantilever. (**a**) Schematic depiction of a quasi-spherical adsorbate on a cantilever vibrating at the resonance frequency of the second flexural vibration mode. (**b**) Opposite effects of the mass and stiffness of the adsorbate on the resonance frequency of the cantilever. (**c**) FEM simulation of the distribution of the bending strain 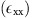 normalized to the maximum value 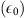 for different contact areas between the adsorbate and the cantilever surface. The shown cross-section plane corresponds to the thickness-length plane of the cantilever.

During the steady-state motion of the cantilever, the average rates of kinetic energy and potential energy must be equal. This equality provides the resonance frequencies of the system and allows an easy understanding of the effect of particle adsorption. When a mass with no stiffness is added to the resonator, the frequency of the resonator must decrease, to keep constant the average rate of kinetic energy. Conversely, the adsorption of a massless particle with stiffness induces an increase of the potential energy of the system due to the energy cost associated to the bending of the particle and thus the frequency must increase, to keep the balance between the average rates of kinetic and potential energies. The mass effect on the resonance frequency increases when adsorption takes place at regions along the beam with higher amplitude (higher kinetic energy), whereas the stiffness effect increases in the regions where the beam undergoes higher changes of curvature during vibration (higher potential energy). Figure 2a,b schematically illustrate the effect of adsorbate adsorption on the resonance frequency of a cantilever. The fractional change of the resonance frequency for the *n*th vibration mode when an analyte lands on the beam can be expressed as the sum of the mass and stiffness contributions^14^,^20^,


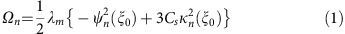


where 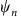 is the mode shape, *ξ*_0_ is the longitudinal adsorption position normalized to the length of the beam, *λ*_m_=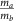 is the mass ratio of the adsorbate to the beam, 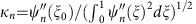 is the normalized curvature of the mode shape and *C*_s_ is referred here to as adsorbate stiffness factor given by,


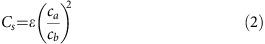


where *c*=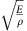 is the one-dimensional speed of sound in the solid, *E* is the Young’s modulus and *ρ* is the density, and the subscripts *a* and *b* refer to the adsorbate and the cantilever, respectively. The dimensionless parameter *ε* includes (i) the linear and quadratic terms of the relative increase of flexural rigidity that become significant for relatively thick and stiff adsorbates^21^, and (ii) the ratio between the effective volume of the adsorbate where the strain is confined and the actual volume of the adsorbate^14^. This last term is smaller than unity, as the bending stress exerted by the beam on the adsorbate is partly screened by the free surface of the adsorbate. The parameter *ε* depends on the shape of the adsorbate and the contact area between the adsorbate and the cantilever, which depends on the adhesion energy, effective elastic modulus and size of the adsorbate^14^. In general, *ε* must be numerically computed by the finite element method (FEM). Figure 2c shows FEM simulations of the bending strain distribution in the beam and in a quasi-spherical adsorbate for different contact areas. As predicted by Euler–Bernoulli beam theory, the strain in the beam increases linearly along the thickness direction. However, this behaviour is not observed in the adsorbate due to the screening effect of the free surface. The simulations show how the mechanical coupling between the beam and the adsorbate increases with the contact area.

### GNP measurements and inverse problem algorithm

We start our experiments by analysing whether the stiffness of 100 nm-sized GNPs can induce a detectable effect on the resonance frequency of nanomechanical resonators. This is perhaps the most challenging test for our method, as the mechanical coupling between the nanoparticles and the microcantilever is rather small due to the reduced contact area and the large surface-area-to-volume ratio of the nanoparticles. We use low-stress silicon nitride microcantilevers with nominal dimensions of 50 × 15 × 0.1 μm (Fig. 1b). The resonance frequencies of the first three flexural vibration modes are of ∼50, 320 and 860 kHz, respectively (Supplementary Fig. 2), and are simultaneously tracked by three digital phase-locked loops (PLLs). The measurement of at least three vibration modes is required to decouple the effect of the adsorption position, mass and stiffness.

**Figure 3:**
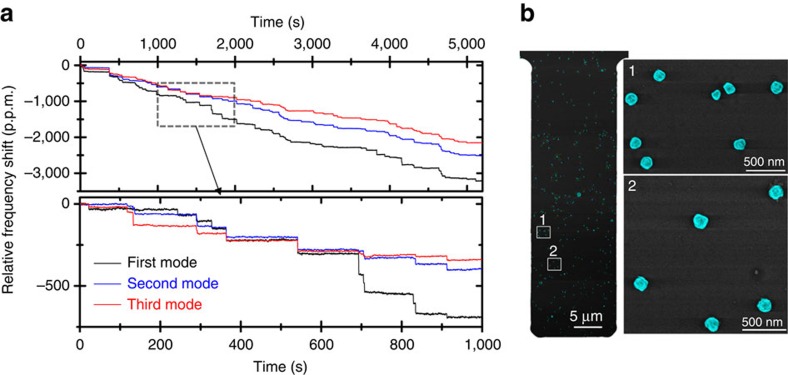
Nanomechanical spectrometry of 100 nm GNPs. (**a**) Real-time record of the resonance frequencies of the first three vibration modes of the microcantilever during nanoparticle adsorption. Quasi-instantaneous jumps are simultaneously observed in the frequencies at the instant in which a single nanoparticle lands on the microcantilever. The total experiment record is shown at the top and a fraction (from *t*=1,000 to *t*=2,000 s) is shown at the bottom. (**b**) At the left, an SEM plan view image of the whole cantilever after the experiment. The adsorbed GNPs are shown in false colour (cyan). The cantilever regions labelled as **1** and **2** are zoomed at the right.

Figure 3a shows a real-time record of the fractional changes of the first three resonance frequencies during ESI of the GNPs. Simultaneous and sporadic jumps in the frequencies are observed with an average rate of two events per minute. The distribution of the GNPs on the cantilever after the experiment was characterized by scanning electron microscopy (SEM; Fig. 3b). The Allan deviations of the first three resonance frequencies at the used acquisition time (70 ms) are 6.0, 2.3 and 1.0 p.p.m., respectively (Supplementary Fig. 3)^4^,^17^.

The analysis of data begins by selecting the time-correlated frequency jumps that fulfill (1) the frequency change occurs in a time faster than the acquisition time, (2) the fractional frequency shift is at least two times the Allan deviation and (3) the frequency jumps occur in at least two vibration modes. For each jump in the frequency, we build the joint probability density function that gives the probability that a jump occurs with fractional frequency changes in the first *n* modes given by 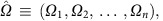



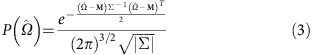


where ***M*** is the mean vector and ∑ is the covariance matrix given by,


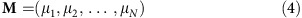



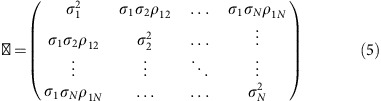


where *ρ*_*ij*_ is the correlation between modes *i* and *j*; *μ*_*i*_ and *σ*_*i*_ are the experimental mean and s.d. of the relative frequency shift of *i*th mode. The use of equation (3) is justified, because the frequency fluctuations in our experiments follow a normal distribution and therefore the joint probability density function must be a multinormal distribution^22^.

The resolution of the inverse problem consists of finding the trio of values (*ξ*_0_, *λ*_m_, *C*_s_) that maximizes the joint probability density function. Mathematically, this is equivalent to finding the values that minimize the following functional


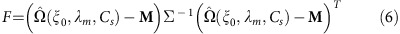


**Figure 4:**
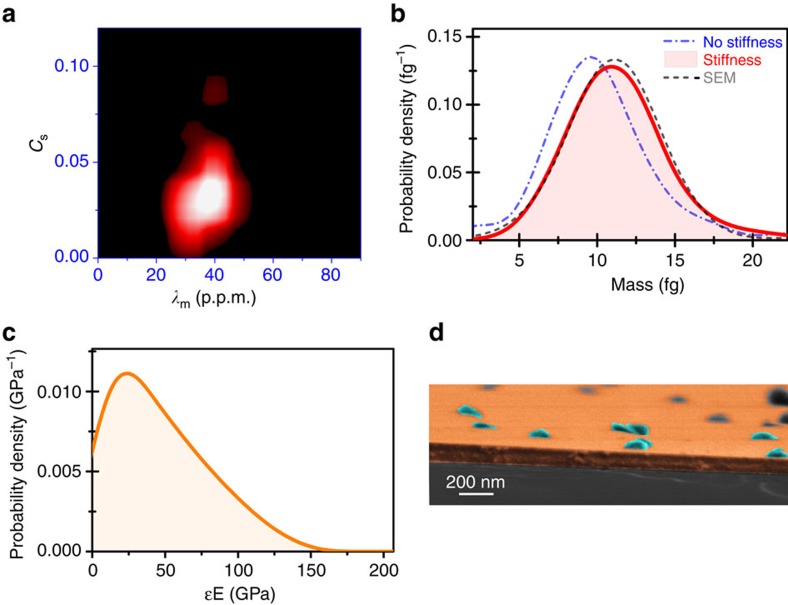
Experimental determination of the mass and stiffness of the nanoparticles. (**a**) Two-dimensional probability density (a.u., intensity colour bar) of the values of the mass ratio and stiffness factor for 120 GNP adsorption events. (**b**) Mass spectra of the nanoparticles neglecting (blue line) and taking into account (red line) the stiffness effect. The distribution of the nanoparticle mass based on high-resolution SEM measurements is also plotted (black dashed line). (**c**) Spectrum of the effective Young’s modulus of the GNPs (*εE*_*a*_). (**d**) SEM image of a region of the cantilever side. The nanoparticles adopt a flattened shape, maximizing the contact area with the cantilever.

We apply the inverse problem method to the time-correlated frequency jumps, (Ω_1_, Ω_2_, Ω_3_), obtained for each GNP landing on the microcantilever to obtain the position, mass and stiffness of the nanoparticles. The derived nanoparticle positions were in good agreement with the positions obtained by dark-field microscopy characterization of the cantilever (Supplementary Fig. 4). Figure 4a shows the two-dimensional probability distribution of the values of the mass ratio and stiffness factor (*λ*_m_, *C*_s_) for 120 events. It is noteworthy that as long as the vibration mode shapes are known, the calculation of these parameters does not require previous knowledge on the cantilever properties (dimensions, density and Young’s modulus). We assume that the vibration mode shapes are described by Euler–Bernoulli beam theory^21^. We find that the mean and s.d. of these orthogonal parameters (*λ*_m_, *C*_s_) are (39.8±13.2 p.p.m., 0.031±0.023). To obtain accurate data on the mass and stiffness of the nanoparticles, the density and Young’s modulus of the cantilever must be calibrated. We harness the well-known effect of the hydrodynamic forces on the frequency response of cantilevers at environmental pressure^23^,^24^. Thus, the resonance frequency and quality factor of the cantilever were measured from the frequency spectra of the Brownian fluctuations of the cantilever in air. Next, we plugged these values into the models of the frequency response of cantilevers immersed in viscous fluids, to derive the spring constant and mass of the cantilever^23^,^24^. SEM was used to measure the exact dimensions of the cantilever for accurate quantification of the Young’s modulus and density of the cantilever, giving 241±22 GPa and 3374±94 kg m^−3^, respectively. The calibration method is detailed in the Supplementary Discussion.

After the calibration of the cantilever, we calculate the probability density of the mass and effective Young’s modulus given by *εE*_*a*_ of the GNPs (Fig. 4b,c). The mass of the nanoparticles is 11.6±3.8 fg (Fig. 4b). If we neglect the stiffness of the nanoparticles, the minimization of the functional (equation (6)) is not so optimal and the mass decreases by 11%, to 10.3±3.7 fg. We carefully calibrate the mass of the nanoparticles by SEM (Fig. 4b) and by AFM (Supplementary Fig. 5). Analysis of 260 nanoparticles by SEM provides a mass of 11.6±5.7 fg, whereas analysis of 215 nanoparticles by AFM also gives very similar value of 11.4±2.8 fg. Both values are in very close agreement with the mass values obtained in the nanomechanical spectrometry by applying our inverse problem algorithm. It becomes clear that the stiffness effect must be accounted for accurate mass measurements. The calculations provide an effective Young’s modulus of 43±32 GPa (Fig. 4c). This value is of about half of the bulk Young’s modulus of gold. Strikingly, the stiffness effect is much higher than expected, if one takes into account that the spherical geometry and small size of the GNPs should provide a rather small mechanical coupling. We analyse the interface between the nanoparticle and the cantilever surface by SEM (Fig. 4d). The images show the nanoparticles significantly flattened on the cantilever, which may be related to the high adhesion energy and the ESI process. AFM characterization confirms this effect: the height of the nanoparticles is 59.0±7.8 nm, that is, a 60% of the original diameter. FEM simulations, where the shape of the nanoparticles on the cantilever is mimicked, provides an effective Young’s modulus of about 55 GPa in consistency with our results (Supplementary Fig. 6). The broad distribution found in the effective Young’s modulus is related to large heterogeneity in the GNP shape and contact area between the nanoparticle and the cantilever.

### Mass and stiffness spectrometry of whole intact bacteria

We apply the nanomechanical spectrometry technique for accurate and high-throughput measurement of the mass and stiffness of individual bacterial cells. The relevance of this application is multifold. First, characterization of microorganisms is essential for rapid diagnosis and targeted treatment of infection. Most of the techniques study the average properties of populations, ignoring the large heterogeneities between individual cells. Our technology enables high-throughput analysis of individual microorganisms based on two orthogonal coordinates, the dry mass and the stiffness. Both parameters provide insights on how the structural conformation, pathological properties and mechanical properties are related to each other^25^. Dry mass is generally estimated by electron microscopy analysis or optical interferometry; however, these measurements are indirect and require assumptions about the material properties^26^. On the other hand, the most widely used method to measure the mechanical properties of biological entities has been so far nanoindentation with the cantilever/tip assembly of an AFM. However, the AFM throughput is still a limiting factor. Here we use our nanomechanical spectrometer to characterize *E. coli* DH5α cells.

**Figure 5:**
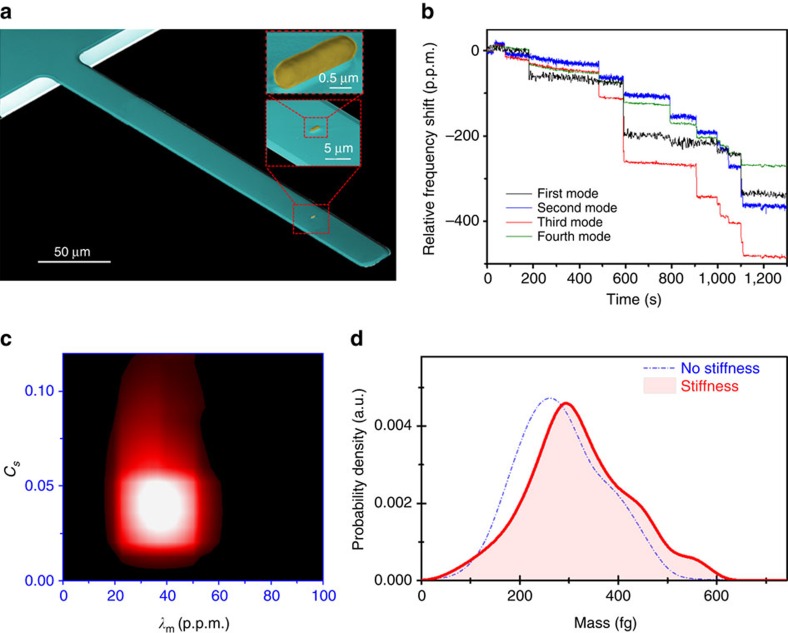
Experimental determination of the mass of the *E. coli* bacteria. (**a**) SEM image of a cantilever with an *E. coli* cell delivered by ESI. The insets show closer views of the whole intact bacterium. (**b**) Real-time record of the fractional shifts of the resonance frequencies of the first four vibration modes of the microcantilever during bacteria adsorption. The time-correlated frequency jumps correspond to adsorption events of single bacterial cells. (**c**) Two-dimensional probability density (a.u., intensity colour bar) of the mass ratio and stiffness factor for 189 bacteria adsorptions. (**d**) Mass spectra of the *E. coli* bacteria neglecting (blue dashed line) and taking into account (red line) the stiffness effect.

Figure 5a shows a SEM image of a bacterium delivered by ESI onto a microcantilever. AFM and SEM morphological analysis of the bacterial cells that arrive to the cantilevers provides a length of 1.87±0.52 μm and a diameter of 0.49±0.05 μm, in close agreement with the dimensions of the native structure. In these experiments, we use silicon nitride cantilevers with nominal length of 200 μm, width of 20 μm and thickness of 0.560 μm. In this case, the resonance frequencies of the first four flexural vibration modes of the cantilever were tracked by PLLs. The modes have initial frequencies of ∼15, 94, 260 and 510 kHz, respectively (Supplementary Fig. 7). The Allan deviations at the used acquisition time (20 ms) are typically 0.82, 0.10, 0.02 and 0.03 p.p.m., respectively (Supplementary Fig. 8). As each bacterium lands on the microcantilever, quasi-instantaneous jumps on the eigenfrequencies are produced (Fig. 5b). We apply our inverse problem algorithm to the correlated fractional frequency jumps to obtain the mass ratio and stiffness factor of the bacterial particles for a total of 189 events (Fig. 5c). The density and Young’s modulus of the cantilever was calibrated following the same procedure used for the nanoparticles (Supplementary Discussion). The obtained dry mass of the bacterial cells is of 318±95 fg that agrees with the values of dry mass estimated by transmission electron microscopy and suspended microchannel resonators^26^,^27^ (Fig. 5d). When the effect of the stiffness is not accounted, the obtained mass values decrease ∼10%. The broad distribution in mass comes from the heterogeneity in the cell population.

**Figure 6:**
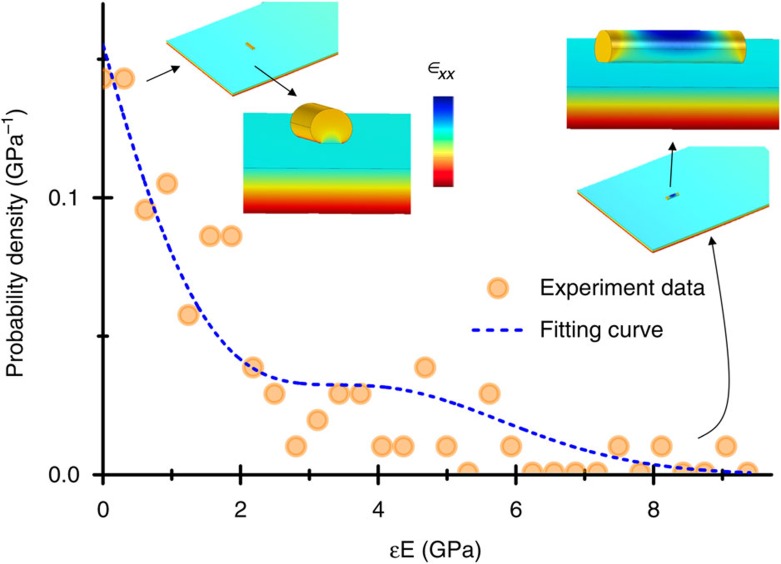
Experimental determination of the effective Young’s modulus of the *E. coli* bacteria. Probability density distribution of the effective Young’s modulus of the *E. coli* bacteria. The lower effective Young’s moduli are related to events in which the long axis of the bacterial cells is approximately transversal to the long cantilever beam axis. Conversely, the higher values of effective Young’s moduli correspond with adsorption events in which the long axes of the bacteria and the cantilever are approximately aligned. The distribution of the bending strain 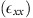 in the cantilever and the bacterial cell are shown for both orientations.

The determination of the bacteria stiffness requires knowledge of the density. We estimate the density of the dry bacteria from the mass calculations and the volume obtained from the AFM and SEM images, being 900±120 kg m^−3^. Figure 6 shows the probability density of the estimated effective Young’s modulus of the bacteria cells (symbols). In contrast with the behaviour of the nanoparticles, the distribution of the effective Young’s modulus is broad and it exhibits a characteristic exponential decay. We relate this behaviour to the rod-like shape of the bacteria cells that induces anisotropy in the stiffness effect on the resonance frequencies. We apply a recently developed theoretical model that provides an analytical formula for the stiffness effect of rod-like analytes on the resonance frequencies of nanomechanical resonators^14^. Briefly, the contact zone between the bacterium and the cantilever is a strip of length, *L*_*a*_ and width 2*a*. When the long axes of the bacterium and the cantilever are aligned, the cantilever bending stress is efficiently transmitted to a significant part of the bacterium volume with little dependence on the contact area (Fig. 6, insets at the right). The strain exponentially decays to zero near the bacterium ends with a characteristic length given by the bacterium diameter. When the long axis of the bacterium is transversally oriented to the beam length, the stress exerted by the cantilever along the contact width is significantly screened by the free surface of the bacterium (Fig. 6, insets at the left). In this case, the contact width plays a critical role and thereby the amount of bending strain within the adsorbate scales up with the contact area. The dimensionless parameter *ε* for rod-like adsorbates is given by,


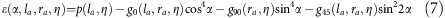


where *α* is the angle between the long axes of the bacterium and the beam, *l*_*a*_ is the ratio between the length and the bacterium diameter, *r*_*a*_ is the ratio between the contact’s width, 2*a*, and bacterium diameter and *η* is the ratio between the bacterium diameter and the beam thickness. The functions *g*_0_(*l*_*a*_,*r*_*a*_,*η*), *g*_90_(*r*_*a*_,*η*), and *g*_45_(*l*_*a*_,*r*_*a*_,*η*) are explicitly given in the Supplementary Discussion. The effect of the bacterial cell orientation produces a probability density function that approximately follows *PDF*(*ε*)=(*a*_0_+*a*_1_*ε*+*a*_2_*ε*^2^)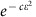, where the constants *a*_0_, *a*_1_, *a*_2_ and *c* are obtained by Monte-Carlo simulations (Supplementary Fig. 9 and Supplementary Discussion). We fit our results to this equation (line in Fig. 6), which allows obtaining the Young’s modulus of the dried bacterial cells, giving a value of 4.2±1.0 GPa. These values compare well with the values obtained by AFM indentation experiments, which range between 4 and 6 GPa (Supplementary Fig. 10). These results show the potential of nanomechanical spectrometry for making quantitative measurements of the Young’s modulus of biological systems.

In conclusion, we demonstrate that nanomechanical spectrometry can be used to characterize the mass and stiffness of intact micrometre- and nanosized analytes. Ignoring the effect of the stiffness can lead to a significant underestimation of the mass, particularly evident in the case of ultrathin cantilevers^20^,^28^. The capability to describe the analytes that arrive to the resonator by two orthogonal coordinates, the mass and the stiffness, clearly enhances the selectivity of nanomechanical spectrometry and it opens the door to relevant biomedical applications. The important role of mechanical properties in biological processes and in pathogenic disorders is becoming increasingly clear. The technology presented here shows great promise for high-throughput characterization of the stiffness in addition to the mass of large biological complexes near their native conformation, a goal that is beyond the capabilities of conventional mass spectrometers.

## Methods

### Nanomechanical spectrometer system

Supplementary Fig. 11a shows a photograph of our nanomechanical spectrometer prototype. The set-up comprises three chambers with decreasing pressure. The top chamber is at ambient pressure, its height is of 8 cm and it contains the ESI needle. The middle chamber is at 10 torr, its height is 12.5 cm and it contains the heated capillary. The heated capillary has an inner diameter of 400 μm and a length of 11.4 cm. The temperature is set to 200 °C and the pressure is kept by a four-stage diaphragm pump (Vacuubrand GmbH, Germany). A skimmer with an orifice of 100 μm connects the middle chamber with the bottom chamber that is at 0.1 torr and houses the microcantilever resonator; its height is 19.5 cm. The microcantilever is placed on a XYZ micro-nanoposition system (Attocube Systems AG, Germany). The pressure in this chamber is kept by a two-stage oil-sealed rotatory pump (Oerlikon Leybold Vacuum GmbH, Germany). The inlet of the vacuum pump is connected just below the resonator holder, to produce a straight-down flow. The ESI needle is fabricated in polyether ether ketone and is connected to the sample solution by a fluidic capillary inject system (Supplementary Fig. 11b). The sample solution is in a 2 ml microtube (Eppendorf, Germany) inside a sample container subject to a pressure of ≈50 mbar, to make the solution flow through the fluidic capillary to the ESI tip needle. The inner diameter of the ESI needle is 63 μm. A high voltage (4–5 kV) is applied to the solution, to form the Taylor’s cone with the subsequent production of charged micro-droplets. The chamber is connected to ground and the case of the heated capillary to 20 V. A charge-coupled device camera (Dino-Lite, Taiwan) is used to image the needle tip and to ensure the correct Taylor’s cone formation. Photographs of the ESI needle before and after the application of the high voltage are shown in Supplementary Fig. 11c,d, respectively. Supplementary 11e shows the formed Taylor’s cone. The distance between the ESI needle tip and the heated capillary is 5 mm. The ESI system (Electrospray ES-3020) was purchased from IONER, Spain. The laser diode used for measuring the cantilever displacement was purchased from Schäfter Kirchhoff GmbH, Germany. The wavelength is 658 nm, the output power 100 μW, the spot diameter 4 μm and the working distance 5.4 cm. The quadrant photodetector that measures the laser beam deflection is from Hamamatsu, Japan. The photocurrent of the upper and lower halves of the quadrant photodetector is amplified by two low-noise transimpedance amplifiers (DHPCA-199 from FEMTO Messtechnik GmbH, Germany). The differential voltage signal is connected to a digital lock-in amplifier (model HF2LI-PLL from Zurich Instruments AG, Switzerland) that enables fast frequency tracking of several vibration modes by digital PLLs.

### Sample preparation

The 100 nm-sized GNPs (Sigma Aldrich, USA) are in citrate buffer solution. After centrifugation of a microtube with 1 ml of the GNP solution (8,000 r.p.m., 10 min, 25 °C), 950 μl of the supernatant was removed and 950 μl of Milli-Q water was added to the microtube. This process was repeated three times. In the last washing step, the GNPs were resuspended in Milli-Q water with 0.5% of Tween 20. The concentration of this GNP solution was measured by using a BioSpectrophotometer (Eppendorf). The concentration of the GNP solution used in the experiments was of 3 × 10^9^ GNPs per ml.

*E. coli* DH5α was kindly supplied by Dr Jesús Mingorance from the Microbiology Department of Hospital La Paz (Madrid, Spain). We used the strain at its stationary phase. Thus, 10 ml of Luria-Bertani broth (Sigma-Aldrich, USA) were inoculated with 50 μl of a stationary phase culture and *E. coli* was grown overnight at 37 °C under agitation before its use. To avoid the formation of any debris from the culture medium, the cells were harvested by centrifugation at 4,400 r.p.m. during 25 min at 20 °C and resuspended in Milli-Q water. This process was repeated three times. Finally, the cells were resuspended in 50% isopropyl alcohol/Milli-Q water. The concentration was adjusted to 10^9^ cell per ml.

### Data availability

All data of this study are available from the authors upon reasonable request.

## Additional information

**How to cite this article:** Malvar, O. *et al*. Mass and stiffness spectrometry of nanoparticles and whole intact bacteria by multimode nanomechanical resonators. *Nat. Commun.* **7,** 13452 doi: 10.1038/ncomms13452 (2016).

**Publisher's note:** Springer Nature remains neutral with regard to jurisdictional claims in published maps and institutional affiliations.

## Supplementary Material

Supplementary Information Supplementary Figures 1-11, Supplementary Discussion and Supplementary References.
