# Supplementary material for: Mass and stiffness spectrometry of nanoparticles and whole intact bacteria by multimode nanomechanical resonators
Source: Nat Commun. 2016 Nov 11;7:13452. doi: 10.1038/ncomms13452 (PMC5476793; doi:10.1038/ncomms13452)
Supplement: Supplementary Information — Supplementary Figures 1-11, Supplementary Discussion and Supplementary References. [file ncomms13452-s1.pdf]

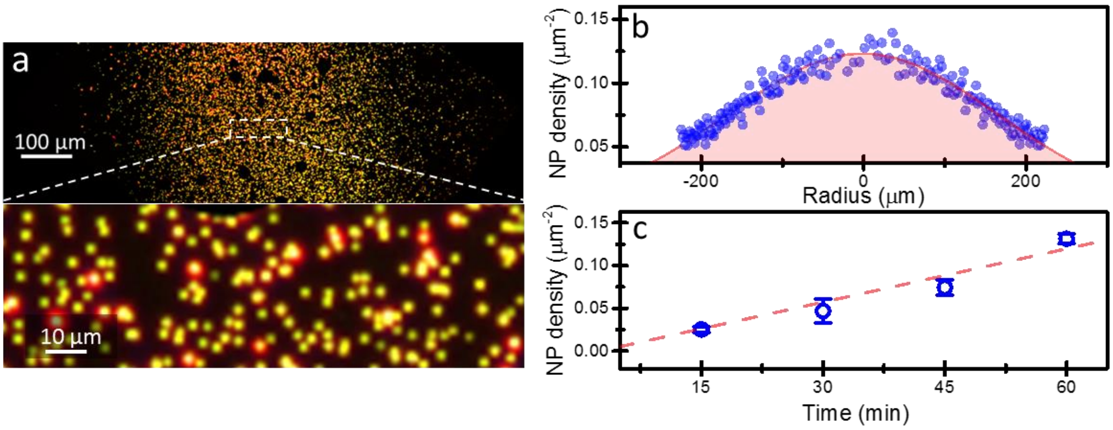

Supplementary Figure 1: Capture efficiency. (a) Dark-field optical images of gold nanoparticles (GNPs) on a Si substrate located at the cantilever holder of the nanomechanical spectrometer after electrospray ionization during 60 min. (b) Surface density of nanoparticles as a function of the radius for a nebulization time of 60 min. (c), Surface density of nanoparticles at the ion beam center as a function of the exposure time (symbols). The line represents a linear fit to calculate the flow rate of nanoparticles at the cantilever chamber.

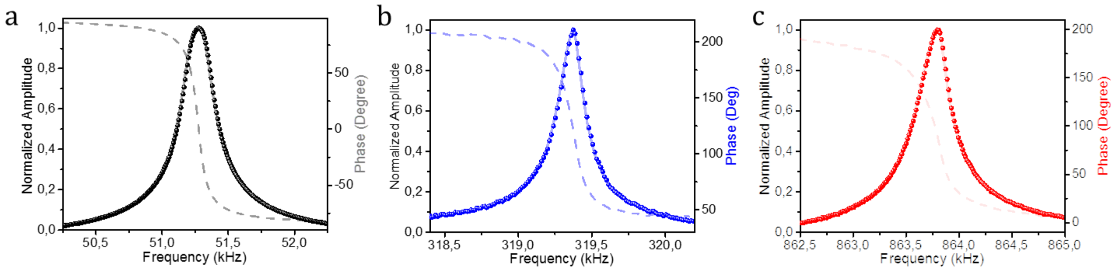

Supplementary Figure 2: Frequency spectra of the first three eigenmodes of a microcantilever used for nanomechanical spectrometry of the gold nanoparticles. The vacuum pressure was 0.1 torr and the cantilever was mechanically driven by a piezoelectric actuator placed underneath the cantilever chip. These measurements were carried out for several cantilevers providing the following values for the first three eigenfrequencies: 49.1±2.2 kHz, 306±14 kHz and 833±31 kHz. The corresponding quality factors were 170±5, 1270±40 and 2000±60.

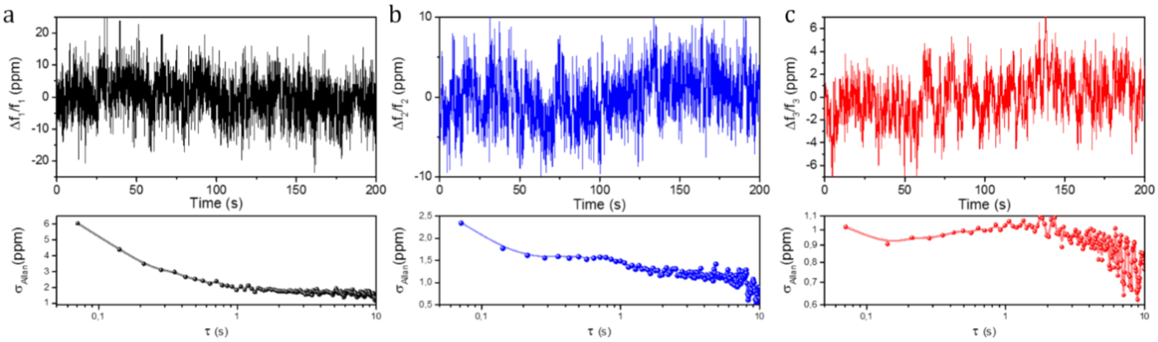

Supplementary Figure 3: Frequency Stability and Allan deviation of a microcantilever used for nanomechanical spectrometry of the gold nanoparticles. Record of the fractional frequency fluctuations (top) and the corresponding Allan deviation (bottom) for the first, (a), second (b) and third (c) vibration modes. The Allan variance, $\sigma_{Allan}(\tau)$,is defined as the variance over time in the measured frequency of a source, each measurement averaged over a time interval τ, with zero-dead time between measurement intervals.

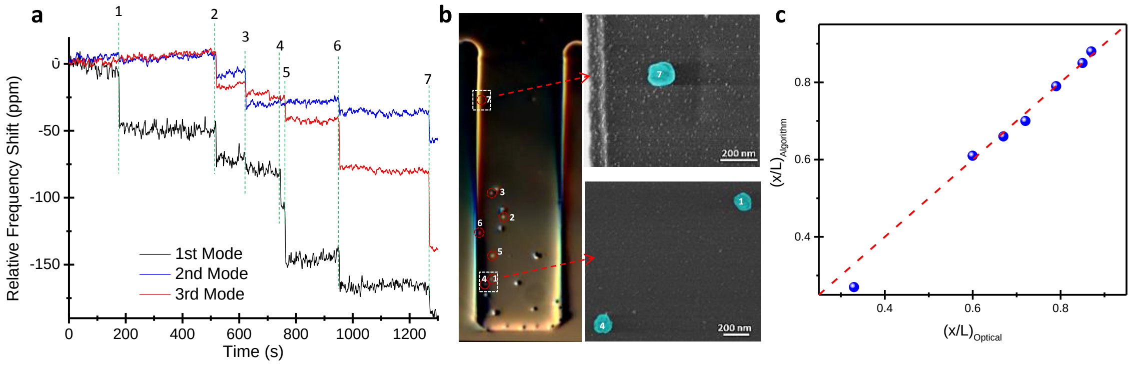

Supplementary Figure 4: Validation of the of the inverse problem algorithm. (a) Real-time record of the resonance frequencies of the first three vibration modes of a microcantilever during gold nanoparticle adsorption. The observed seven frequency jump events are numbered for identification. (b) Post-processing dark field optical microscopy image resulting of the subtraction of the images after and before the experiment (left). Big bumps are impurities produced during the cantilever fabrication and are not labelled. Seven nanoparticles were identified on the cantilever surface in consistency with the observed eigenfrequency jumps. The nanoparticles are numbered in order to correlate with the events identified in graph (a) using the inverse problem algorithm (main text). We confirmed that the identified dark-field spots were real nanoparticles by carrying out SEM characterization of two areas of the cantilever marked in the optical image (right). (c) Comparison between the positions measured by the optical microscope and the positions obtained applying our inverse problem algorithm (symbols). The line depicts the '*perfect*' correlation. The positions are normalized to the length of the cantilever.

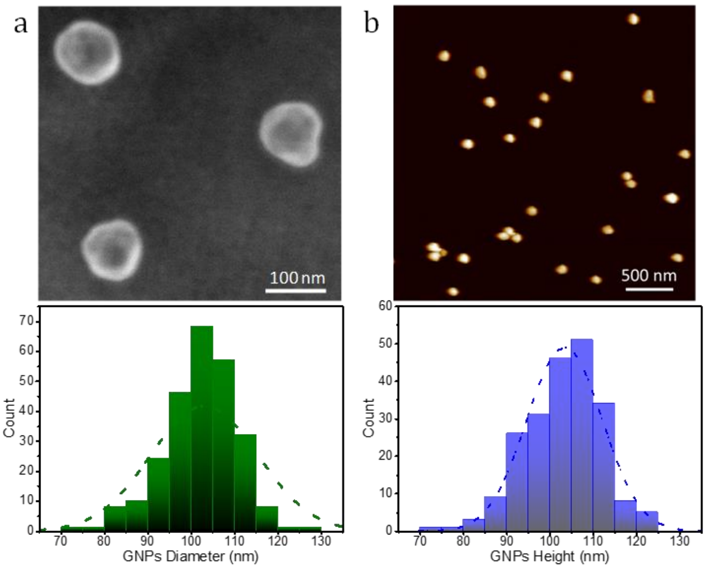

Supplementary Figure 5: SEM and AFM characterization of the gold nanoparticles (GNPs). (a) At the top, a typical SEM image used for measuring the GNP diameter. At the bottom, diameter histogram of 260 GNPs. The histogram is fitted to a normal distribution that provides a diameter of (103±13) nm. (b) At the top, typical AFM image for measuring the GNP height using the PeakForce Tapping mode. The AFM was previously calibrated with a grating (TGS1 from NT-MDT, Russia). At the bottom, height histogram of 215 GNPs. The histogram is fitted to a normal distribution that provides a height of (103±9) nm.

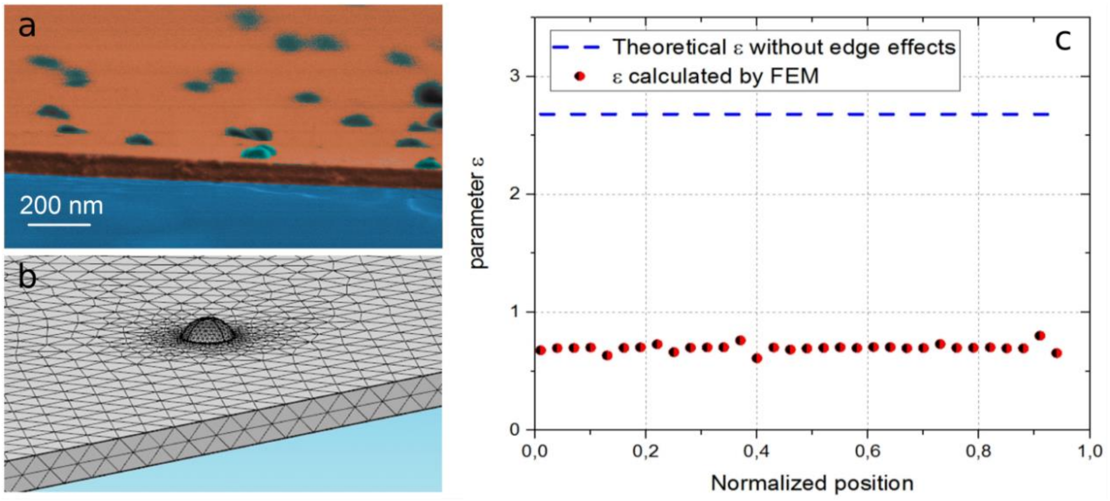

Supplementary Figure 6: FEM simulation of stiffness of the adsorbed gold nanoparticles. (a) SEM image in false color of some of the gold nanoparticles adsorbed on the cantilever surface. (b) FEM model of a gold nanoparticle on the microcantilever. The nanoparticle is modelled as a spherical cap with height of 60 nm (close to the value obtained by AFM, 59.0±7.8 nm) and a radius of 66 nm (in order to match the volume of the nanoparticles, 5.24×10^5 nm^3). (c) FEM calculation of the parameter ε by applying the inverse problem algorithm to the resonance frequency shifts induced by the nanoparticle for several positions along the cantilever. The positions are normalized to the cantilever's length. The FEM simulations provides ε=0.7±0.03.

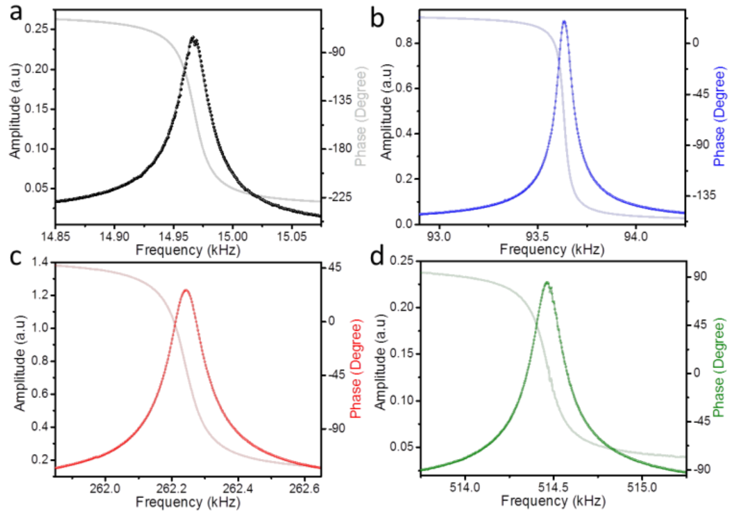

Supplementary Figure 7: Frequency spectra of the first four eigenmodes of a microcantilever used for nanomechanical spectrometry of the bacteria. The vacuum pressure was 0.1 torr and the cantilever was mechanically driven by a piezoelectric actuator placed underneath the cantilever chip. These measurements were carried out for several cantilevers providing the following values for the first three eigenfrequencies: 14.8±0.2 kHz, 93.5±1.2 kHz, 261.5±1.5 kHz and 511.5±2.5 kHz. The corresponding quality factors were 370±10, 680±10, 1504±10 and 2000±20.

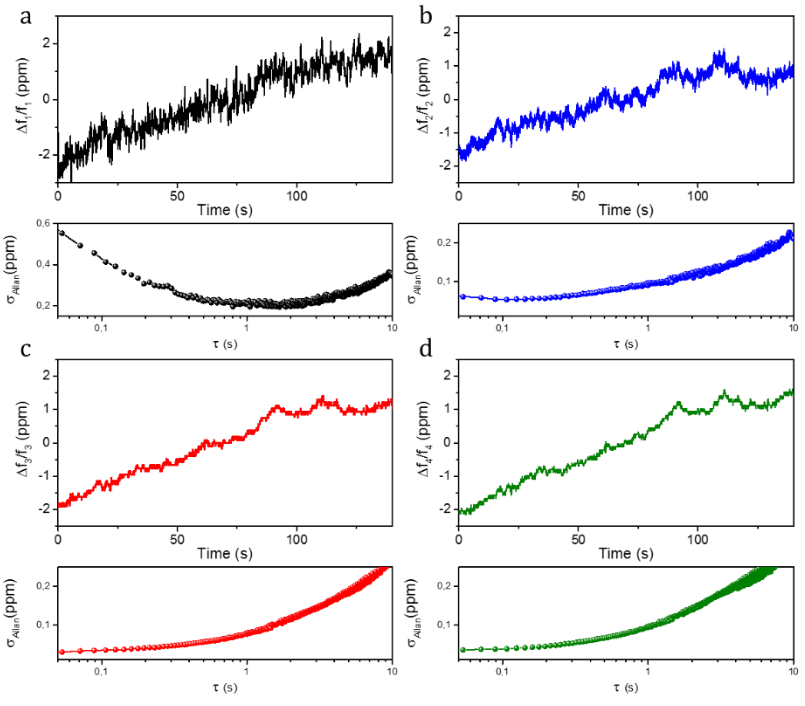

Supplementary Figure 8: Frequency Stability and Allan deviation of a microcantilever used for nanomechanical spectrometry of the bacteria. Record of the fractional fluctuations of the eigenfrequencies of the microcantilever (top) and the corresponding Allan deviation (bottom) of the first (a), second (b), third (c) and fourth (d) mode, respectively. The Allan variance, $\sigma_{Allan}(\tau)$,is defined as the variance over time in the measured frequency of a source, each measurement averaged over a time interval τ, with zero-dead time between measurement intervals.

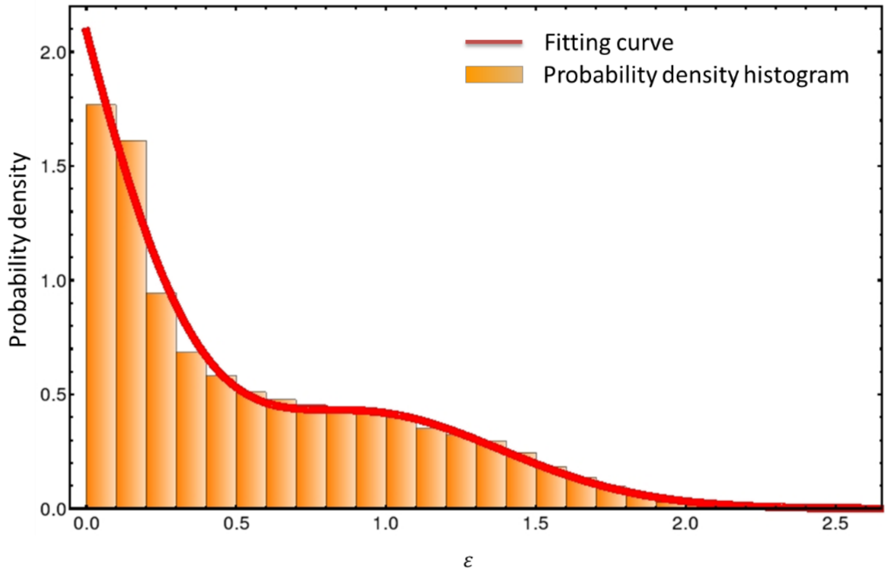

Supplementary Figure 9. Monte-Carlo simulation of the probability density function of ε for *E. coli* bacteria. See related text in the Supplementary Discussion.

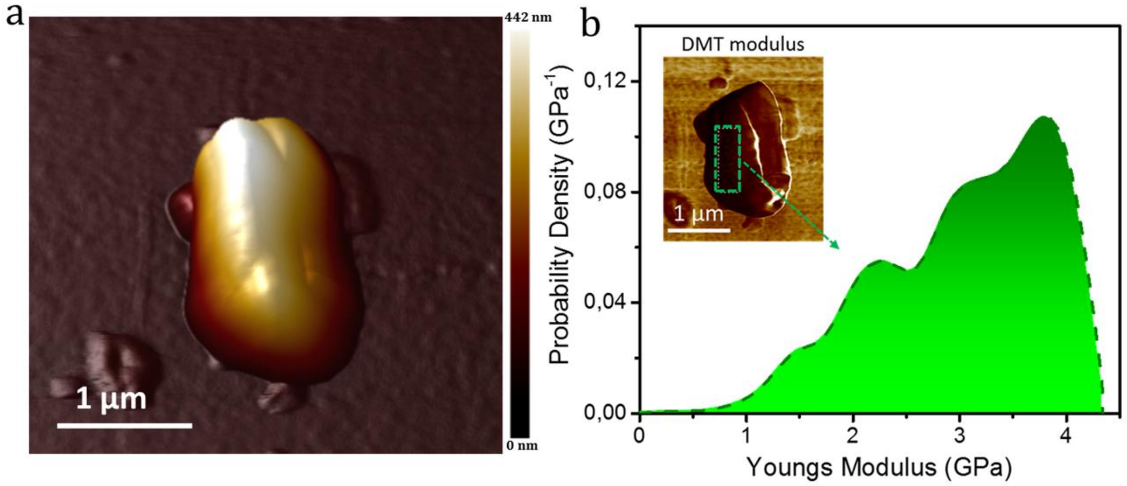

Supplementary Figure 10: AFM characterization of Young´s modulus of a *E. coli* bacteria. (a) AFM image of a *E. coli* bacterium obtained by nebulization with the ESI system. The height of the shown bacterial cell is 460±20 nm. The used AFM is a Nanoscope IV Multimode AFM (Bruker) in the PeakForce Tapping mode. We use a stiff cantilever probe (TAP525, Bruker, USA) with a nominal spring constant of 200 N/m in order to ensure the indentation of the dry *E. coli*. The tip radii were estimated by SEM after the experiments, ranging 200-300 nm. The spring constant was calibrated by using the thermal tune method. (b) Young's modulus distribution of the marked area in the image inset. We apply forces of 1-2 µN. The Young's modulus is obtained by applying the DMT(Derjagin, Muller, Toropov) model. We measured different *E. coli* previously nebulized with the ESI system. The obtained Young´s modulus in different areas of different *E. Coli* cells range between 3 and 5 GPa. In (b) the Young's modulus distribution results into 3.6±0.8 GPa.

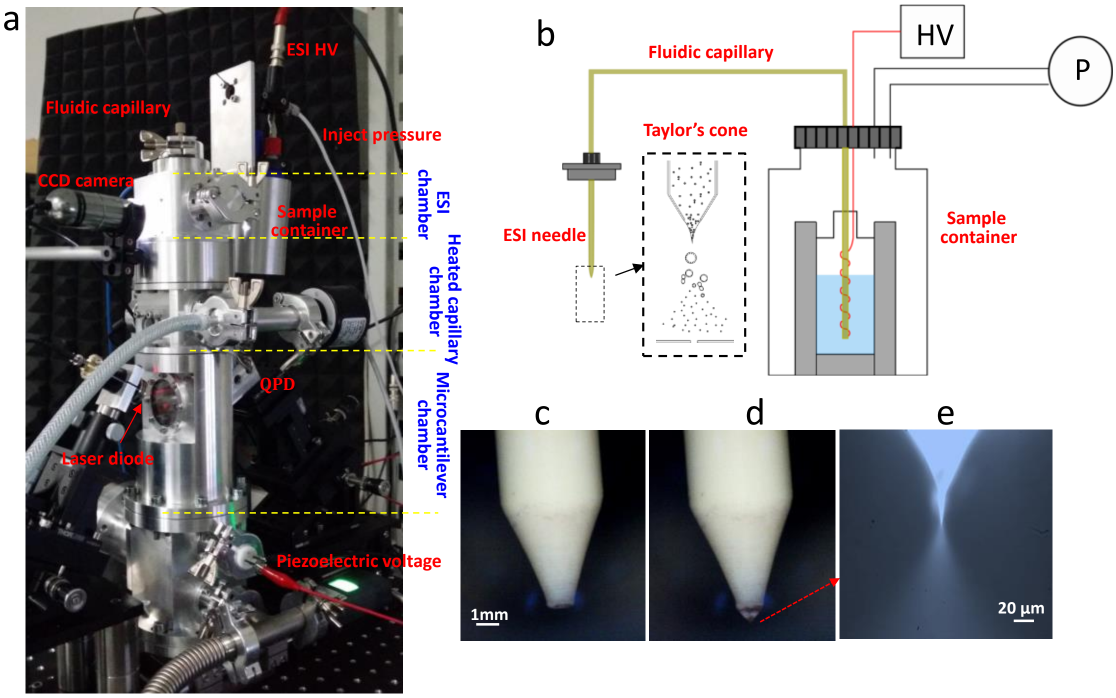

Supplementary Figure 11: Nanomechanical spectrometer. (a) Photograph of the nanomechanical spectrometer prototype. (b) Schematic depiction of the fluidic capillary inject system and ESI source used to produce the charged species stripped of solvent. Photographs of the ESI needle before and during the application of 4.5 kV are shown in (c) and (d) respectively. (e) Microscopic image of the Taylor's cone formed in our system.

Supplementary Discussion

Capture Efficiency of GNPs

The capture efficiency of the gold nanoparticles defined as the ratio between the number of gold nanoparticles (GNPs) that reach the cantilever surface and the number of GNPs that leave the peek needle was estimated as follows. The GNP flow rate was measured just below the ESI source and also at the cantilever position by placing a Si substrate at both positions and *ex-situ* counting the nanoparticles that arrive the surface by a home-made dark-field optical microscope equipped with an ultrahigh-definition cooled color camera (DS-RI1, Nikon, Tokyo, Japan). The silicon substrates were previously cleaned with Piranha solution (3 H_2SO_4 : 1 H_2O_2).The GNPs on the Si substrates were analyzed by an algorithm written in Matlab (MathWorks®, USA) that identifies the nanoparticles and calculates the radial surface density of GNPs, which approximately follows a Gaussian distribution. Integrating the surface density for all the radii, we estimate that 1564 GNPs/s are emitted by the ESI needle. The flow rate of nanoparticles at the cantilever chamber is of 3.7±0.3 nanoparticles/s (Supplementary Fig. 1). This is 0.24% of the nanoparticles emitted by the ESI needle. The nanoparticle beam at the cantilever chamber exhibits very low divergence, ≈0.2 deg, that enables to collect most of the nanoparticles in a reduced area with radius of about 300 μm (Supplementary Fig. 1). Accounting for the ratio of the cantilever plan view area to the ion beam cross-section, we estimate an overall collection efficiency of $\approx 10^{-5}$.

Calibration of the density and Young's modulus of the cantilevers

The density and the Young's modulus of the cantilevers were calculated from the frequency and quality factor in air taking advantage of the well-known hydrodynamic effects described by Sader's theory[1,2]. The resonant frequency and quality factor of the cantilever vibrating in air are given by,

$$\frac{\omega_R}{\omega_{vac}} = \left(1 + \frac{\pi \rho b}{4\rho_c h}\Gamma_r^f(\omega_R)\right)^{-1/2} \quad (1)$$

$$Q = \frac{\frac{4\rho_c h}{\pi \rho b} + \Gamma_r^f(\omega_R)}{\Gamma_i^f(\omega_R)} \quad (2)$$

where ω_R is the radial resonant frequency in the fluid, ρ is the density of the fluid, $\rho_c$is the density of the cantilever, b and h are the width and thickness of the cantilever respectively, $\Gamma_r^f(\omega_R)$ and $\Gamma_i^f(\omega_R)$ are the real and imaginary part of the hydrodynamic function described in reference[1,2] and ω_{vac} is the radial resonant frequency in vacuum given by,

$$\omega_{vac} \approx \frac{3.516h}{L^2}\left(\frac{E_c}{12\rho_c}\right)^{1/2} \quad (3)$$

where E_c is the Young's modulus of the cantilever. We experimentally determine the resonance frequency and quality factor from the frequency spectra of the thermal fluctuations of the microcantilevers in air. We substitute these values and the cantilever dimensions obtained by SEM in supplementary equations (1)-(3) to obtain the density and Young's modulus of the cantilever.

For the cantilevers used for nanomechanical spectrometry of gold nanoparticles, we obtained a density and a Young's modulus of 3374±94 kg/m^3 and 241±22 GPa, respectively. For the cantilevers used for the *E. coli* measurements, we obtained a density and a Young's modulus of 4127±103 kg/m^3 and 171±13 GPa, respectively.

Theory of the bending stiffness of *E. coli* bacteria

Since the shape of the *E. coli* bacteria is cylindrical, we use the model described in reference[3] for the calculation of parameter ε,

$$\varepsilon(\alpha, l_a, r_a, \eta) = p(l_a, \eta) - g_0(l_a, r_a, \eta)\cos^4\alpha - g_{90}(r_a, \eta)\sin^4\alpha - g_{45}(l_a, r_a, \eta)\sin^2 2\alpha \quad (4)$$

where α is the angle between the long axes of the bacteria and the cantilever beam, l_a is the ratio between the length and the diameter of the bacteria, r_a is the ratio between the contact width and the bacteria diameter and η is the ratio between the diameter of the bacteria and the thickness of the cantilever. The functions $g_0(l_a, r_a, \eta)$, $g_{90}(r_a, \eta)$, and $g_{45}(l_a, r_a, \eta)$ are explicitly given by,

$$g_0(l_a, r_a, \eta) = \frac{B_0(r_a, \eta)\tanh\left(\frac{3\, p(r_a, \eta) l_a}{B_0(r_a, \eta)}\right)}{3\lambda_a} \quad (5)$$

$$g_{90}(r_a, \eta) = \frac{B_{90}(r_a, \eta)\tanh\left(\frac{p(r_a, \eta) r_a}{B_{90}(r_a, \eta)}\right)}{3 r_a} \quad (6)$$

$$g_{45}(l_a, r_a, \eta) = a_0(l_a, r_a, \eta)(a_1(l_a, r_a, \eta) + a_1(r_a, l_a, \eta) + a_2(l_a, r_a, \eta)) \quad (7)$$

where

$$a_0(l_a, r_a, \eta) = \frac{4p(r_a, \eta)\sinh\left(\frac{B_{45}(\eta)l_a}{2}\right)^2 \sinh\left(\frac{B_{45}(\eta)r_a}{2}\right)^2}{B_{45}(\eta)^2 l_a r_a \left(1-\cosh(B_{45}(\eta)l_a)\cosh(B_{45}(\eta)r_a)\right)^2} \quad (8)$$

$$a_1(l_a, r_a, \eta) = B_{45}(\eta)l_a \coth\left(\frac{B_{45}(\eta)l_a}{2}\right)\left(\cosh(B_{45}(\eta)r_a)\cosh\left(\frac{B_{45}(\eta)l_a}{2}\right)^2 - 1\right) \quad (9)$$

$$a_2(l_a, r_a, \eta) = -\sinh(B_{45}(\eta)l_a)\sinh(B_{45}(\eta)r_a) \quad (10)$$

where

$$p(\eta, r_a) = \frac{1}{3}[3 + 6\eta + 3.75\eta^2 - (2.54\eta + 1.27\eta^2)r_a - (0.94\eta + 1.31\eta^2){r_a}^2] \quad (11)$$

$$B_0(\eta, r_a) = 9.33 + 25.18\eta + 10.83\eta^2 - (11.25 + 35.86\eta + 12.88\eta^2)r_a - (5.83 + 17.19\eta + 2.13\eta^2){r_a}^2 \quad (12)$$

$$B_{90}(\eta, r_a) = 2.53 + 7.10\eta + 11.56\eta^2 - (0.42 + 3.5\eta + 10\eta^2)r_a \quad (13)$$

$$B_{45}(\eta) = 1.07 - 0.09\eta + 0.29\eta^2 \quad (14)$$

Supplementary equation (4) is modified with respect to reference 3 in order to account for the moderate-small l_a of bacteria. Also, for the sake of simplicity, the effect of the Poisson's ratio of the cantilever has been neglected since this effect is very small.

From SEM, optical and AFM images, we have observed that the bacteria are adsorbed with a random angle α, they have a parameter l_a following a normal distribution of mean 3.81 and a standard deviation of 1.06, the parameter η has a mean value of 0.88 with a standard deviation of 0.07. Using these values, we generate 100k adsorptions using supplementary equation (4) and construct the corresponding probability density function (Supplementary Fig. 9). The parameter r_a was randomly varied between 0.1 and 0.6.

We found that the probability density function can be fitted by the simple equation,

$$PDF(\varepsilon) = (a_0 + a_1\varepsilon + a_2\varepsilon^2)e^{-c\varepsilon^2} \quad (15)$$

where $a_0 = 2.085$, $a_1 = -4.968$, $a_2 = 4.617$ and $c = 1.440$.

REFERENCES

1. Sader, J. E. Frequency response of cantilever beams immersed in viscous fluids with applications to the atomic force microscope. *J. Appl. Phys.* **84**, 64-76, (1998).

2. Van Eysden, C. A. & Sader, J. E. Frequency response of cantilever beams immersed in viscous fluids with applications to the atomic force microscope: Arbitrary mode order. *J. Appl. Phys.* **101**, 044908 (2007).

3. Ruz, J., Tamayo, J., Pini, V., Kosaka, P. & Calleja, M. Physics of Nanomechanical Spectrometry of Viruses. *Sci. Rep.* **4** (2014).
